# Corilagin: A Novel Antivirulence Strategy to Alleviate *Streptococcus pneumoniae* Infection by Diminishing Pneumolysin Oligomers

**DOI:** 10.3390/molecules27165063

**Published:** 2022-08-09

**Authors:** Qiushuang Sheng, Xiaoning Hou, Nan Wang, Minda Liu, Haoyu Zhu, Xuming Deng, Xiaoying Liang, Gefu Chi

**Affiliations:** 1 State Key Laboratory for Zoonotic Diseases, Key Laboratory for Zoonosis Research, Ministry of Education, College of Veterinary Medicine, Jilin University, Changchun 130062, China; 2 Department of Internal Medicine, University of South Florida, Tampa, FL 33620, USA; 3 The Affiliated Hospital of Inner Mongolia Medical University, Hohhot 010010, China

**Keywords:** corilagin, pneumolysin, antivirulence, molecular docking, *Streptococcus pneumoniae*

## Abstract

Pneumolysin (PLY) is a significant virulence factor of *Streptococcus pneumoniae* (*S. pneumoniae*), able to break through the defense system of a host and mediate the occurrence of a series of infections. Therefore, PLY as the most ideal target to prevent *S. pneumoniae* infection has received more and more attention and research. Corilagin is a tannic acid that exhibits excellent inhibition of PLY oligomers without bacteriostatic activity to *S. pneumoniae*. Herein, hemolytic activity assays, cell viability tests and western blot experiments are executed to evaluate the antivirulence efficacy of corilagin against PLY in vitro. Colony observation, hematoxylin and eosin (H&E) staining and cytokines of bronchoalveolar lavage fluid (BALF) are applied to assess the therapeutic effect of corilagin in mice infected by *S. pneumoniae*. The results indicate the related genes of corilagin act mainly via enrichment in pathways associated with pneumonia disease. Furthermore, molecular docking and molecular dynamics simulations show that corilagin might bind with domains 3 and 4 of PLY and interfere with its hemolytic activity, which is further confirmed by the site-directed mutagenesis of PLY. Additionally, corilagin limits PLY oligomer production without impacting PLY expression in *S. pneumoniae* cultures. Moreover, corilagin effectively relieves PLY-mediated cell injury without any cytotoxicity, even then reducing the colony count in the lung and the levels of pro-inflammatory factors in BALF and remarkably improving lung lesions. All the results demonstrate that corilagin may be a novel strategy to cope with *S. pneumoniae* infection by inhibiting PLY oligomerization.

## 1. Introduction

The infectious disease caused by *S. pneumoniae* is one of the most serious public health problems in the world, especially, the mortality rate is approximately one million in children younger than 5 years mainly due to meningitis, bacteremia, acute otitis media, sinusitis, bacteremic pneumonia etc. [1,2]. Antibiotics are still the main strategy for clinical treatment. However, the drug resistance of *S. pneumoniae* is becoming more and more serious due to the widespread use of antibiotics, making the resulting infections increasingly difficult to cure.

PLY protein, with a molecular weight of 53 kDa, belongs to the family of cholesterol-dependent cytolysins (CDCs), which is the essential virulence factor of *S. pneumoniae* that invades the host [3]. PLY localizes to cholesterol on the host cell membrane and subsequently undergoes a conformational change to form oligomers that exert pore-forming toxicity, leading to cell lysis. The protein structure consists of four asymmetric domains that are slightly curved rather than spherical. Domain 1 at the N-terminal includes a negative charge [4]. Upon insertion into the membrane, the negative charge contributes to the orientation of the oligomers [5,6,7]. Domain 4 is connected to the process of oligomerization, which is pushed upwards. Then, domain 3 is driven towards the inner surface toward the developing ring; thereafter, the helical region of domain 3 inserts into the membrane [8]. Moreover, during the infection process, PLY is mainly responsible for alveolar edema, hemorrhage, and can even impair the function of phagocytes or immune cells. Accordingly, the PLY-deficient mutants of *S. pneumoniae* are attenuated to induce pneumocyte injury and the inflammatory response [9,10,11]. Consequently, PLY may be a promising target on which to screen inhibitors, which might result in the discovery of a potential important therapeutic strategy for the treatment of pneumonia infections.

Corilagin (1- acyl-3, 6-hexahydroxy bibenzoyl glucose) has mainly been found in Longan, *Phyllanthus urinaria L* and *Phyllanthus emblica Linn* [12,13] and shows excellent efficacy treating anticancer, antioxidant, antiinfection and anti-cardiovascular diseases including hypertension, atherosclerosis, congestive heart failure and ischemic cardiomyopathy [14,15,16,17,18]. However, corilagin’s defense of lung injury is reported primarily in this paper. A series of experiments both in vitro and in vivo indicate that corilagin is an ideal inhibitor when combined with PLY for the prevention of PLY-mediated *S. pneumoniae* infection.

## 2. Results

### 2.1. Corilagin Efficiently Attenuated the Hemolytic Activity of PLY

**Figure 1:**
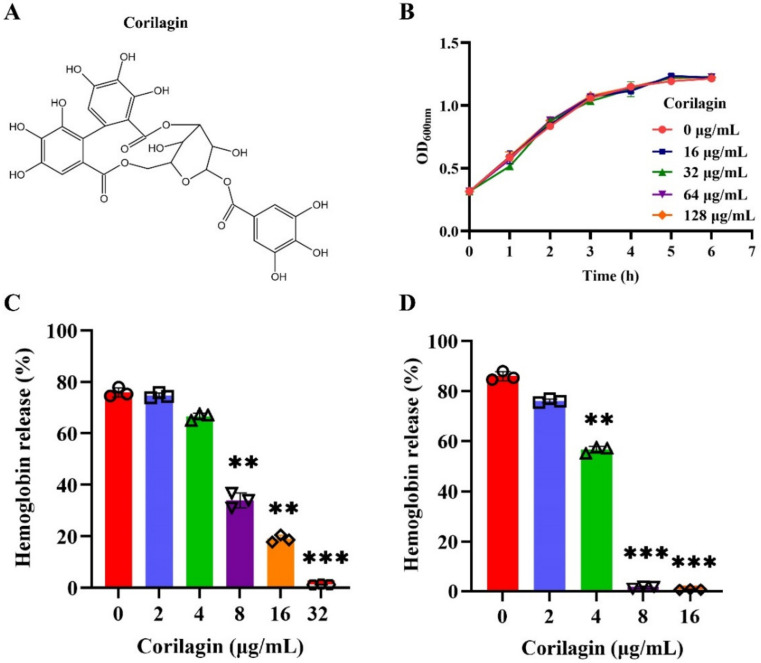
Corilagin restrained the hemolytic efficacy of PLY. (**A**) The chemical structure of corilagin. (**B**) Growth curve of *S. pneumoniae* challenged with corilagin at different concentrations (0, 16, 32, 64 and 128 μg/mL). The efficacy of corilagin against the hemolysis both in *S. pneumoniae* supernatants (**C**) and purified PLY (**D**), ** *p* < 0.01, *** *p* < 0.001.

According to the above analysis, *S. pneumoniae* was used to induce lung infection in order to evaluate the activity of corilagin. The chemical structure of corilagin (Figure 1A) presented as a polyphenolic tannic acid; therefore, antibacterial activity analysis was executed first. Unexpectedly, corilagin did not inhibit the growth of *S. pneumoniae* in the indicated concentrations and time (Figure 1B). Conversely, PLY, as the important virulence product of *S. pneumoniae,* was significantly sensitive to corilagin. As Figure 1C depicts, the hemolytic efficacy of *S. pneumoniae* supernatants declined remarkably from nearly 80% to 2% after being exposed to different concentrations of corilagin. The same result was also represented in purified PLY challenged with corilagin (Figure 1D); compared with no corilagin treatment, the hemolytic activity significantly diminished to 0.5% after treatment with 16 μg/mL corilagin. Therefore, it was confirmed that the inhibition against the hemolytic activity of bacterial supernatants was mainly due to the limitation of PLY’s activity. Taken together, we found an effective inhibitor of PLY’s virulence factor primarily in tannic acid, which might be a promising anti-virulence strategy. 

### 2.2. Corilagin Suppressed the Production of PLY Oligomers

**Figure 2:**
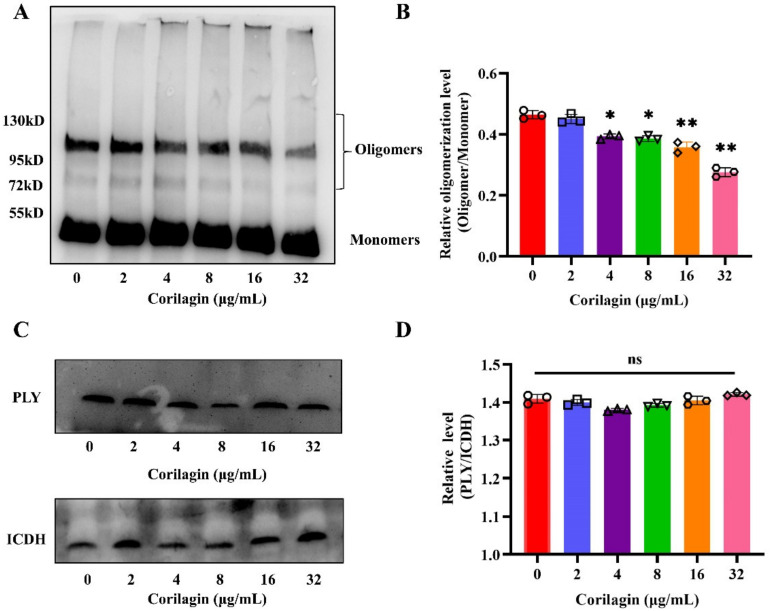
The formation of PLY oligomers was inhibited by corilagin. (**A**) Western blot analysis of purified PLY incubated with different concentrations of corilagin (0, 2, 4, 8, 16 and 32 μg/mL). (**B**) The oligomerization level of PLY was quantitated by ImageJ software (* *p* < 0.05, ** *p* < 0.01). (**C**) PLY expression of *S. pneumoniae* D39 was verified by Western blot assay exposed to various concentrations of corilagin (0, 2, 4, 8, 16 and 32 μg/mL). (**D**) The quantified assay of the expression level of PLY by ImageJ software (ns, no significant difference).

To our knowledge, the PLY-mediated pathogenic process of *S. pneumoniae* in the host depended on oligomer formulation. Therefore, blocking PLY oligomer formation would be an effective targeting strategy to inhibit hemolysis. As the results manifested (Figure 2A), corilagin suppressed PLY oligomerization in a concentration-dependent manner, with excellent inhibition occurring at 32 μg/mL. Moreover, as the oligomers decreased, the intensity of the monomer blot exhibited an upward trend. The ImageJ software was applied to quantify the inhibition effect of hemolysis in which the ratio of oligomer/monomer was obviously diminished at 32 μg/mL compared to no corilagin treatment (Figure 2B). Additionally, the Western blot assay demonstrated that in different concentrations of corilagin co-cultured with *S. pneumoniae,* the expression of PLY protein was not significantly affected (Figure 2C). In contrast to ICDH, the grayscale analysis showed no difference in the relative level of PLY/ICDH (Figure 2D). Taken together, the results above illustrated that corilagin inhibited the hemolytic activity of PLY via repression of the formation of oligomers, without affecting PLY expression.

### 2.3. Corilagin Enhanced the A549 Cell Viability during S. pneumoniae Invasion

**Figure 3:**
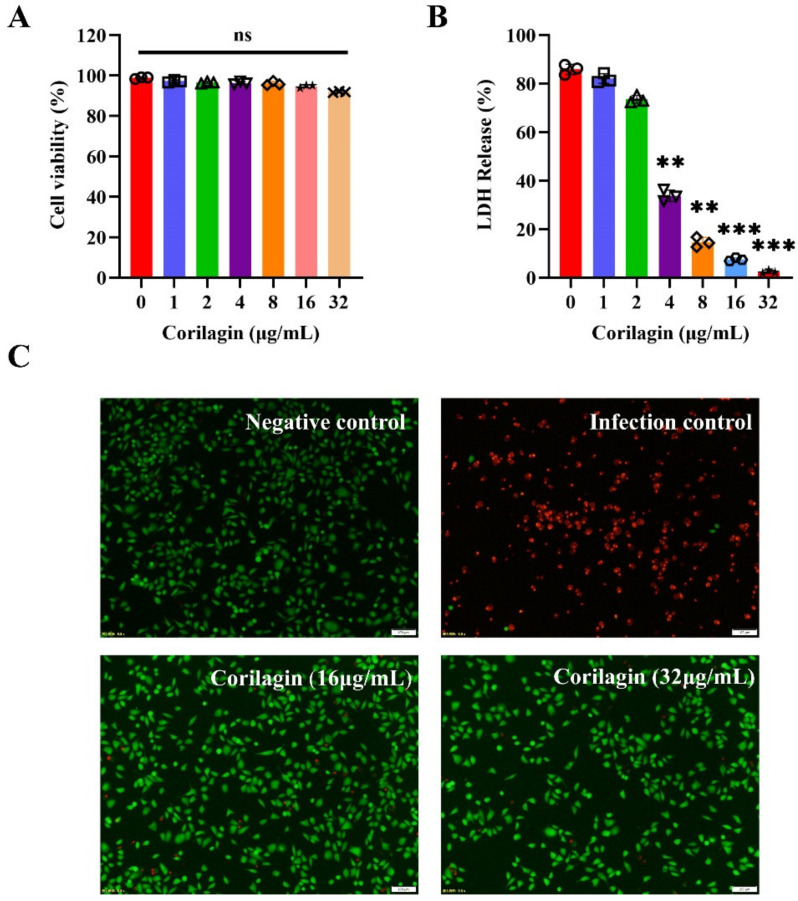
Corilagin relieved PLY-mediated A549 cell damage. (**A**) The cell viability of A549 cells after being treated by corilagin at different concentrations (0, 1, 2, 4, 8, 16 and 32 μg/mL). (ns, no significant difference). (**B**) The LDH release detection of A549 cells incubated with purified PLY with various concentrations of corilagin treatment (0, 1, 2, 4, 8, 16 and 32 μg/mL). ** *p* < 0.01, *** *p* < 0.001. (**C**) The fluorescence images of A549 cells treated with PLY as infection control, corilagin at 16 and 32 μg/mL, respectively, and no treatment as negative control.

As previously reported, PLY has been confirmed to damage many types of cells in the host [19,20,21]. Herein, A549 cells were used to assess the potential protective efficacy of corilagin on PLY-mediated A549 cell injury. Interestingly, A549 cells challenged with corilagin alone displayed excellent cell viability (Figure 3A). This implied that corilagin itself had low toxicity, which facilitated the subsequent study. When the cells co-cultured with purified PLY with or without corilagin, as the LDH data showed (Figure 3B), corilagin can effectively inhibit the damage PLY does to cells. The release of LDH was negatively correlated with the concentration of corilagin. Once the concentration of corilagin reached up to 32 μg/mL, it presented the most prominent cytoprotective effect. Meanwhile, fluorescence images visualized the above conclusions (Figure 3C); the negative control without any treatment exhibited a robust green influence intensity which showed the perfect cell viability. However, in the infection control, the luminous influence intensity for cell death was clearly visualized after PLY was challenged without corilagin treatment. Exhilaratingly, when adding 16 μg/mL or 32 μg/mL of corilagin, the vast majority of cells displayed a predominant viability, especially when treated with corilagin at 32 μg/mL, which fully indicated that corilagin displayed excellent activity against PLY-mediated cell damage (even the cytoprotective function was very obvious).

### 2.4. Corilagin Effectively Prevented Mice from S. pneumoniae Infection

**Figure 4:**
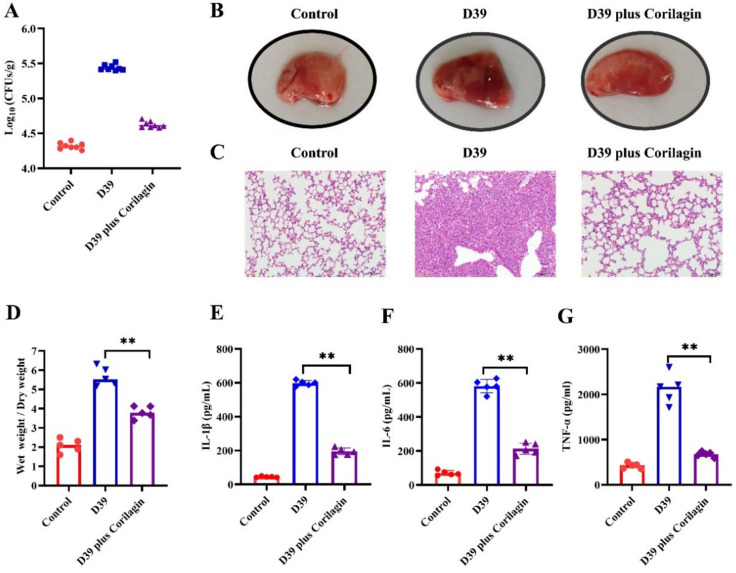
Corilagin effectively protected mice from *S. pneumoniae* D39 infection. The mice were infected with *S. pneumoniae* intranasally and, thereafter, were treated with or without corilagin (50 mg/kg). After 96 h of infection, the bacterial burden of the lung (**A**) was calculated and the photographs of the ocular pathological changes of the lung (**B**) were displayed after the mice died via dislocation. Then, the haematoxylin and eosin staining slices (**C**) presented the histopathological changes of the lung. Then, (**D**) the wet/dry weight ratio of lung tissue and the levels of IL-1β (**E**), IL-6 (**F**) and TNF-α (**G**) in the bronchoalveolar lavage fluid (BALF) were all detected to assess the efficacy of corilagin for *S. pneumoniae*-infected mice. ** *p* < 0.01.

The therapeutic effect of corilagin for infected mice was investigated via nasal inoculation of *Streptococcus pneumoniae* and subcutaneous injection of corilagin solution. As anticipated, the mice in the D39 infection group showed extensive lung tissue damage and extremely high colony counts compared with the control, whereas after adding the corilagin, the colony counts decreased significantly and even the gross lesion was remarkably relieved (Figure 4A,B). In further observations, after being challenged with D39, the alveolar structure of the mouse lung completely deformed with massive inflammatory cell infiltration, which can be clearly recognized on H&E staining slices. Conversely, after corilagin treatment, the histological changes in the infected lung recovered nearly to the level of the control group, with the alveolar structure complete and orderly and no inflammatory cell infiltration visible to the naked eye (Figure 4C). In addition, the ratio of wet weight/dry weight was also significantly declined in the corilagin treated group compared to the D39 infected mice (Figure 4D), which indicated that the corilagin can effectively relieve pulmonary edema and greatly alleviate lung damage. Likewise, the main pro-inflammatory factors (IL1β, IL6 and TNFα) related to inflammatory infections were significantly suppressed in the corilagin treatment group in comparison to the D39 group (Figure 4E–G), which indicated that corilagin can efficiently control the damage of inflammatory infections and reduce the damage to the host due to pathogenic bacteria.

### 2.5. Analysis and Validation of Potential Mechanisms of Action

**Figure 5:**
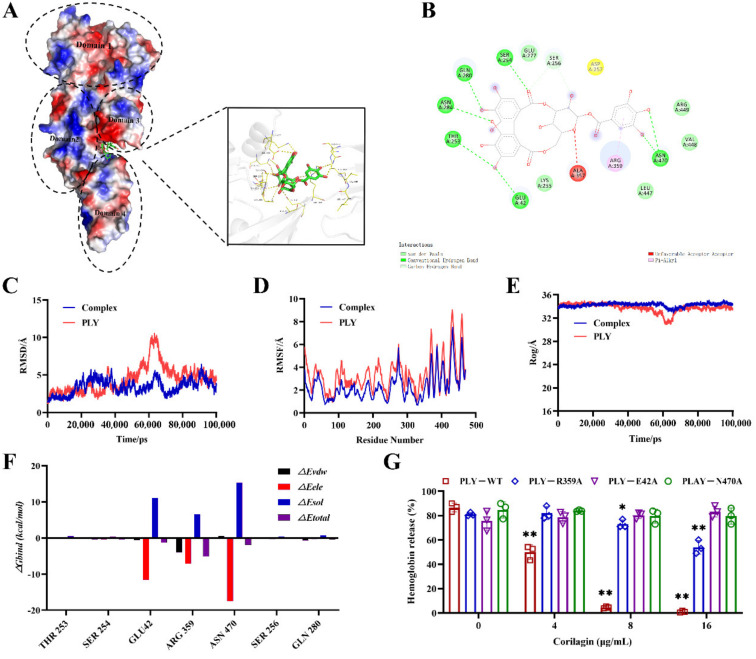
Prediction of binding modes of corilagin to PLY protein using molecular modeling. (**A**) The binding mode of corilagin to PLY on the basis of MD simulation. During the standard MD simulation process, corilagin could strongly embed at the groove between Domain 3 and Domain 4 of PLY. (**B**) Asn 470, Glu 42, Thr 253 and Arg359 residues were the key sites for corilagin binding with PLY. (**C**) RMSD fluctuation of the complex (corilagin−PLY) and PLY throughout the MD simulation was found. (**D**) RMSF curves of the complex and PLY were obtained. (**E**) The Rog curves of the complex and PLY were obtained. (**F**) The binding energies of the complex and PLY were calculated according to an MM−PBSA analysis. (**G**) Mutation of binding sites disrupted the inhibitory effect of corilagin on PLY. * *p* < 0.05, ** *p* < 0.01.

To observe the interacting mechanism of PLY and corilagin, molecular docking and stimulating were carried out to analyze the difference between complexes and PLY alone. As shown in Figure 5, corilagin, due to the multiple hydroxyl groups in the structure, could be accurately embedded into the gap between Domains 3 and 4 in PLY mainly through hydrogen bonding (Figure 5A); this occurred on Gln280, Asn284, Thr253, Glu42, Ser254, Asn470, etc., or via the Pi-Alkyl bond with Arg 359 on the right benzene ring of corilagin (Figure 5B). These bonding interactions were the critical and main force for the stable bonding of small molecules to PLY and this further interfered with the PLY’s function of oligomerization. Furthermore, the root-mean-square deviation (RMSD) curve was relatively stable after 80 ns of the protein without small molecules. After binding to the small molecules, the RMSD curve fluctuated greatly throughout the kinetic process, indicating that the binding of small molecules had a remarkable impact on protein conformational stability (Figure 5C). Meanwhile, we calculated the root-mean-square fluctuations (RMSF) of residues in PLY and complexes; a large flexible complex at the C terminal domain was found in PLY, showing the variability of the four functional regions; the fluctuation profile of the complexes showed similar distributions along the residue index (Figure 5D). This indicated that the new system of complexes still followed the dynamic properties of the original system of PLY. Then, in the radius of the gyration (Rog) curve, we observed the remarkable fluctuations in PLY, although it became stable after 80 ns. In the complexes, the fluctuations of Rog were slowed down, which indicated that the conformation of the PLY protein changed distinctly (Figure 5E). Accordingly, the energy decomposition profiles verified that the residues of Asn 470, Arg 359 and Glu 42 made contact with corilagin mainly via van der Waals and hydrogen bonding interactions (Figure 5F). In order to verify the above predictions of molecular docking and molecular simulations, site-directed mutagenesis of PLY was carried out to produce E42A, R359A and N470A according to their energy decomposition profiles. As Figure 5G displays, corilagin almost lost its inhibitory effect on PLY hemolysis when the 42-position glutamate and 470-position aspartate of the PLY protein were mutated, indicating that the binding of this site severely affected the oligomerization of PLY; however, when the mutation appeared on the 359-position arginine, the drug retained a slight inhibitory effect on PLY as the concentration increased. All the results indicated that the binding of corilagin with the PLY protein mainly affected the oligomerization of PLY through the 42-position glutamate and 470-position aspartate, further reducing the virulence of PLY.

## 3. Discussion

*S. pneumoniae,* a pneumococcus, is a leading cause of community-acquired pneumonia (CAP), a kind of invasive pneumococcal disease (IPD), and this bacterium has become a matter of worldwide visibility due to its limited and dissatisfactory therapeutic options in clinical applications [9,22,23]. Despite the assertion that the pneumococcal conjugate vaccine (PCV) or antibiotics are effective regimens against *S. pneumoniae* bacteria and able to reduce the incidence of CAP or IPD in children and adults, the complicated serotype and serious resistance of the bacteria severely diminishes the clinical therapeutic approach [24,25]. Therefore, the identification of potent, alternative, antimicrobial strategies to cope with this urgent need is of great importance.

Although its multiple biofunctions have been demonstrated extensively, the potential efficacy of corilagin against pneumococcal diseases has not been fully explored. In this study, a series of phenotypic experiments were conducted to evaluate the effect of corilagin on *S. pneumoniae* in vitro. To our knowledge, this was the first study to exploit a tannic acid to treat infectious diseases caused by *S. pneumoniae.* Importantly, we found that the addition of corlilagin significantly reduced the hemolytic activity of PLY from *S. pneumoniae* via the prevention of the oligomerization of the PLY monomer in vitro, which was the most crucial virulence factor to form a pore on the bacterial cell membrane. This was further assessed through molecular ducking, in which the formation of stable hydrogen bonds was very conducive to the binding of PLY and corilagin, thus influencing the conformation of Domain 3 and Domain 4 to reduce the oligomerization of PLY, and then inhibiting PLY-mediated hemolytic activity, which is a critical step for pore formation. The site-directed mutagenesis of PLY further demonstrated that corilagin’s ability to reduce the virulence of PLY was mainly attributed to its binding to the 42-position glutamate and 470-position aspartate of the PLY protein, which further affected the oligomerization of PLY. As expected, corilagin significantly reduced lung damage in the in vivo test, demonstrating a lower colony count, wet weight/dry weight ratio and pro-inflammatory factor levels when compared with the infected group, which displayed prominent activity against PLY-mediated damage to the host cell.

## 4. Materials and Methods

### 4.1. Bacterial Strains, Cell Lines and Chemicals

The *S. pneumoniae* strain D39 (NCTC 7466) adopted in this paper was a bestowal from Dr. David E. Briles from the University of Alabama (Birmingham, UK). Todd Hewitt broth with 2% yeast extract (THY) was the ideal culture for D39, which was kept at 37 °C without shaking. A549 cells (human lung epithelial cells) were obtained from the American Type Culture Collection (ATCC, CCL-185, Manassas, VA, USA) and conserved in Dulbecco’s modified Eagle’s medium/high glucose (DMEM; HyClone, Carlsbad, CA, USA), mixing with 1% penicillin–streptomycin (MRC, Madrid, Spain) at a condition of 5% CO_2_ and 37 °C. Corilagin (purity > 98%) was purchased from Shanghai yuanye Bio-Technology Co., Ltd. (Shanghai, China) and dissolved in pure dimethyl sulphoxide (DMSO, Sigma-Aldrich, St. Louis, MO, USA).

### 4.2. Mutant Construction and Protein Expression and Purification

The process of this test was performed mainly according to a previous report [26]. Briefly, site-directed mutagenesis of PLY was carried out using a QuikChange kit (Stratagene, La Jolla, CA, USA) to produce E42A, R359A and N470A with pET28a-PLY as the template (Lab-constructed cryopreserved strains). The primers were PLY-bamhi-F/CTGGGAT GATCCatggcaaataaagcagtaaatg, PLY-XHOI-R/CTGCTCGAGctagtcattttctaccttatcctc, PLY -Glu42-F/5′-tctttcgataacaacaaacgcatcgggtagctgattacc-3′, PLY-Glu42-R/5′-ggtaatcagctacccg atgcgtttgttgttatcgaaaga-3′, PLY-Arg359-R/5′-agactaaggttacagcttacgcaaacggagatttactgctgg- 3′ and PLY-Asn470-R/CTGCTCGAGctagtcTGCttctaccttatcc, respectively. The pET28a-PLY and mutant constructs were transformed into *Escherichia coli* BL21 (DE3) and then overexpressed. The bacteria supernatant was collected for further use. 

### 4.3. Molecular Docking and Molecular Dynamics Simulation

The 3D X-ray structures (PDB code: 4QQA, 2.80 Å) of the PLY protein (GenBank: CP000410.2) were obtained and the SDF structure of corilagin (CID: 73568) was obtained from the PubChem site. To get the docking−binding models for corilagin with 4QQA, AutoDock Vina 1.1.2 was adopted to carry out the docking process of flexible ligands and rigid receptors. Thereafter, the molecular dynamics simulation was performed for the complexed systems. The whole detailed process is depicted in the previous literature [27].

### 4.4. PLY Purification

The PLY sequences were ligated into a pET-28a vector and then purified using a method consistent with previous reports [26].

### 4.5. Haemolysis Assay

The purified PLY and mutant constructs were challenged with corilagin at different concentrations (0, 2, 4, 8 and 16 μg/mL). After incubating for 30 min, an appropriate amount of rabbit blood was added for another 10 min of incubation at 37 °C. Subsequently, the supernatant was measured at 570 nm after centrifugation with 1000× *g* for 1 min using a microplate reader (Tecan, Grödig, Austria). PBS, water and DMSO were used as the negative control, positive control and solvent control, respectively. Correspondingly, D39 was incubated overnight and diluted with 100 times the amount of fresh THY, and then cultured until OD 600 nm of 1.2 following centrifugation to obtain the culture supernatant treated with different concentrations of corilagin (0, 2, 4, 8, 16 and 32 μg/mL) for 1 h at 37 °C. The rest of the steps were the same as mentioned above for the purified PLY.

### 4.6. Growth Curve Test

D39 was cultured overnight and diluted 1:100 with fresh, sterile THY at 37 °C to obtain the bacteria suspension with an optical density of 0.1 at 600 nm. Afterward, the suspension was divided into five equal parts into conical flasks and exposed to corilagin at different concentrations (0, 16, 32, 64 and 128 μg/mL) for continuous incubation at 37 °C; then the OD value of each sample was measured at 600 nm every 30 min for 7 h.

### 4.7. PLY Expression Assessment with Western Blot Assay

The *S. pneumoniae* D39 was cultured overnight and diluted with fresh THY (1:100) and then treated with a series of concentrations of corilagin (0, 2, 4, 8, 16 and 32 μg/mL) for sustained incubation until OD 600 nm of 1.2. The bacterial pellet was collected by centrifugation, resuspended in a Laemmli sample buffer and boiled for 10 min. Isocitrate dehydrogenase (ICDH) was used as an internal reference. Thereafter, 12% SDS-PAGE was used to separate supernatants and then transferred onto a PVDF membrane blocked with 5% skim milk. Subsequently, the membrane was co-incubated with anti-pneumolysin monoclonal antibodies (1:1000, Abcam, Cambridge, UK), detected with HRP-labeled secondary anti-mouse antibodies (1:2000, Proteintech) and visualized with ECL reagent (Thermo scientific, Rockford, IL, USA). Eventually, the blots were observed on an imager (Tanon, Shanghai, China) [28] and the grayscale value was quantified by ImageJ software.

### 4.8. Oligomerization Analysis

Purified PLY was challenged with different concentrations of corilagin (0, 2, 4, 8, 16 and 32 μg/mL) for 1 h of incubation at 37 °C, 5 × SDS-PAGE loading buffer was added and maintained for another 10 min at 50 °C. Then, 6% SDS-PAGE was adopted to separate the suspension using the same protocol of the Western blot assay as described above [29]. 

### 4.9. Cell Viability and Cytotoxicity Assays

According to the manufacturer’s instructions, cell counting kit-8 (CCK-8) (APExBIO, Houston, TX, USA) was applied to assess the cytotoxicity of corilagin. Concisely, A549 cells were seeded into 96-well plates with a density of 2 × 10^4^ and some concentration series of corilagin (0, 1, 2, 4, 8, 16 and 32 μg/mL) were added to cell wells for 5 h of incubation at 37 °C. DMEM was served as the negative control and DMSO as the solvent control. After that, each well was treated with a CCK-8 working solution for another 1 h of incubation at 37 °C. By the end, the 96-well plates were placed on a microplate reader (Tecan) with an absorbance at 450 nm.

The PLY protein was challenged with different concentrations of corilagin (0, 1, 2, 4, 8, 16 and 32 μg/mL) for 30 min at 37 °C and then added into 96-well plates containing A549 cells at a density of 2 × 10^4^ for each well for another 5 h of incubation at 37 °C as previously reported [26]. Thereafter, we used the Cytotoxicity Detection Kit to detect the release level of lactate dehydrogenase (LDH) in the culture supernatant according to the manufacturer’s protocols (Roche, Switzerland).

Subsequently, the cell morphology or viability was assessed via the live/dead cell fluorescent staining as previously depicted [26]. Briefly, purified PLY was exposed to different concentrations of corilagin (0, 1, 2, 4, 8, 16 and 32 μg/mL) for 30 min and then incubated with A549 cells for 5 h. According to the live/dead reagent (Invitrogen, Carlsbad, CA, USA) protocols, the working reagent was added to the cell pellets after centrifugation of the co-culture for 45 min and the imagines were acquired via immunofluorescence microscopy (Olympus, Tokyo, Japan).

### 4.10. Animal Test

Female adult Balb/c mice weighing 18–20 g (6 to 8 weeks old) were used in this study, which were obtained from Liaoning Changsheng Biaotechnology Co., Ltd. (Liaoning, China). All the animal experiments were conducted according to the guidelines of the Animal Care and Use Committee (ACUC) of Jilin University. Before the infection test, the mice were housed in standard conditions with a light/dark cycle (12 h/12 h) and fed normal food and water for 5–7 days.

The D39 with OD600 nm of 0.4 was obtained, after centrifugation (8000× *g*, 10 min), the bacteria pellets were washed three times with PBS and then resuspended to 1 × 10^10^ CFU/mL. Mice were randomly divided into three groups (D39 infected group, corilagin treated group (50 mg/kg) and no treatment group as the control) with 7–8 mice in each group. For the colony count or slices of lung analysis, each mouse was slightly anesthetized with ether; then the bacteria suspension (2 × 10^8^ each mouse) was administrated via the nasal passage. Thereafter, corilagin solution was injected into infected mice at 1 h post infection by subcutaneous administration at 8 h intervals for 3 days, and the control group was injected with PBS according to the same schedule. The bronchoalveolar lavage fluid (BALF) was collected to determine the cytokine levels with ELISA (Biolegend, San Diego, CA, USA) before all the mice were killed by cervical dislocation after 4 days. Partial lung tissue drying was executed to determined wet weight/dry weight ratio and the lung tissue lesions were observed by H&E staining.

### 4.11. Statistical Analysis

The data was displayed as the mean ± SD with at least three biological replicates and then analyzed via the Kruskal–Wallis test as follows: * *p* < 0.05, ** *p* < 0.01, *** *p* < 0.001 and ns (no significant difference).

## 5. Conclusions

For a long time, the role of tannic acid has been ignored. Although it is an important chemical component in herbs, no clinically relevant therapeutic products are available and its efficacy in the field of pneumonia is rarely reported. In this study, we first discovered that corilagin, a tannic acid compound, displayed distinct inhibition to the PLY protein. This novel PLY inhibitor was meaningful as it greatly reduced the pathogenicity of *S. pneumoniae* to the host, which proved a novel strategy of tannic acid against *S. pneumoniae* infection in the field of natural compounds.

## Data Availability

The data that support the findings of this study are available from the corresponding author upon reasonable request.

## Sample Availability

Samples of the compounds that support the findings of this study are available from the corresponding author upon reasonable request.
